# 2-[(1-Oxidopyridin-4-yl)sulfan­yl]benzoic acid

**DOI:** 10.1107/S1600536814009854

**Published:** 2014-05-10

**Authors:** Rodolfo Moreno-Fuquen, Leidy Valencia, Javier Ellena

**Affiliations:** a Departamento de Química – Facultad de Ciencias, Universidad del Valle, Apartado 25360, Santiago de Cali, Colombia; b Instituto de Física de São Carlos, IFSC, Universidade de São Paulo, USP, São Carlos, SP, Brasil

## Abstract

In the title compound, C_12_H_9_NO_3_S, the dihedral angle between the pyridine and benzene rings is 83.93 (7)°. In the crystal, pairs of O—H⋯O hydrogen bonds link the molecules, forming inversion dimers with graph-set notation *R*_2_^2^(22). These dimers are in turn linked by weak C—H⋯O hydrogen bonds along [100], forming *R*_2_^2^(8) rings.

## Related literature   

For a novel synthesis of organic sulfur compounds, see: Moreno-Fuquen *et al.* (2010 ▶); For standard bond-length data, see: Allen *et al.* (1987 ▶). For hydrogen bonding, see: Nardelli (1995 ▶). For graph-set motifs, see: Etter (1990 ▶).
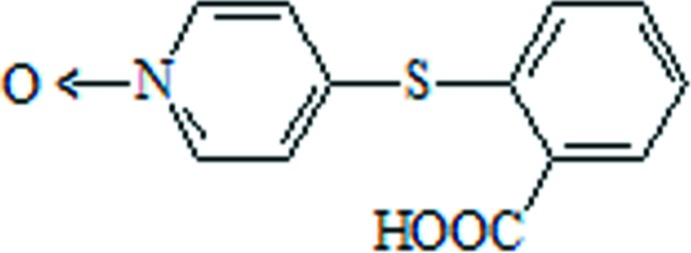


## Experimental   

### 

#### Crystal data  

C_12_H_9_NO_3_S
*M* *_r_* = 247.26
Monoclinic, 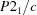

*a* = 8.9894 (8) Å
*b* = 5.7373 (3) Å
*c* = 22.5855 (18) Å
β = 111.348 (4)°
*V* = 1084.92 (14) Å^3^
*Z* = 4
Mo *K*α radiation
μ = 0.29 mm^−1^
*T* = 295 K
0.39 × 0.09 × 0.08 mm

#### Data collection  

Bruker–Nonius KappaCCD diffractometer
Absorption correction: multi-scan (*SADABS*; Sheldrick, 2002 ▶) *T*_min_ = 0.944, *T*_max_ = 0.966
7031 measured reflections
2439 independent reflections
1209 reflections with *I* > 2σ(*I*)
*R*_int_ = 0.063

#### Refinement  

*R*[*F* ^2^ > 2σ(*F* ^2^)] = 0.048
*wR*(*F* ^2^) = 0.132
*S* = 0.96
2439 reflections
154 parameters
H-atom parameters constrained
Δρ_max_ = 0.22 e Å^−3^
Δρ_min_ = −0.27 e Å^−3^

### 

Data collection: *COLLECT* (Hooft, 2004 ▶); cell refinement: *SCALEPACK* (Otwinowski & Minor, 1997 ▶); data reduction: *DENZO* (Otwinowski & Minor, 1997 ▶) and *SCALEPACK*; program(s) used to solve structure: *SHELXS97* (Sheldrick, 2008 ▶); program(s) used to refine structure: *SHELXL97* (Sheldrick, 2008 ▶); molecular graphics: *ORTEP-3 for Windows* (Farrugia, 2012 ▶) and *Mercury* (Macrae *et al.*, 2006 ▶); software used to prepare material for publication: *WinGX* (Farrugia, 2012 ▶).

## Supplementary Material

Crystal structure: contains datablock(s) I, global. DOI: 10.1107/S1600536814009854/lh5700sup1.cif

Structure factors: contains datablock(s) I. DOI: 10.1107/S1600536814009854/lh5700Isup2.hkl

Supporting information file. DOI: 10.1107/S1600536814009854/lh5700Isup3.cml

CCDC reference: 1000546

Additional supporting information:  crystallographic information; 3D view; checkCIF report

## Figures and Tables

**Table 1:** Hydrogen-bond geometry (Å, °)

*D*—H⋯*A*	*D*—H	H⋯*A*	*D*⋯*A*	*D*—H⋯*A*
C11—H11⋯O3^i^	0.93	2.35	3.255 (3)	164
O1—H1⋯O3^ii^	0.82	1.74	2.530 (3)	162

Symmetry codes: (i) 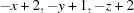; (ii) 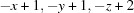.

**Fig. 1.**
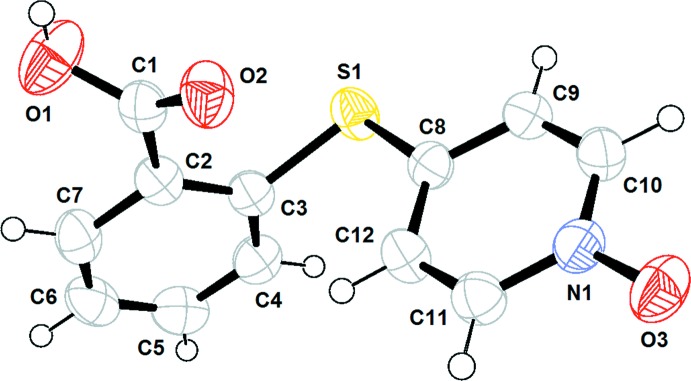
An ORTEP-3 (Farrugia, 2012) plot of (I) with displacement ellipsoids drawn at the 50% probability level. H atoms are shown as spheres of arbitrary radius.

**Fig. 2.**
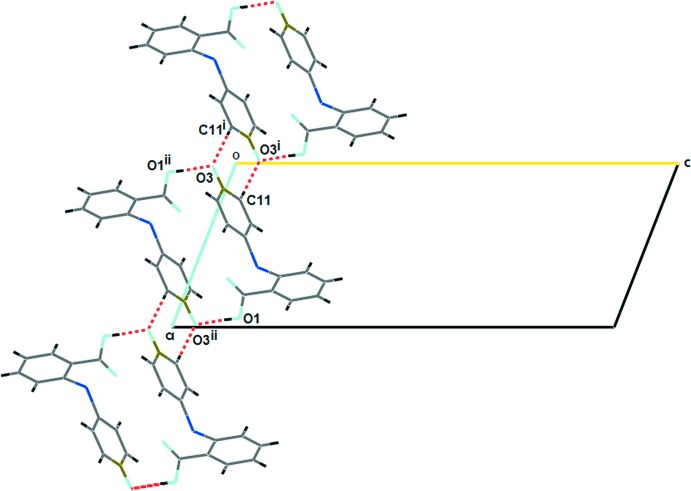
Part of the crystal structure of (I), showing the formation of chains of molecules running along [100]. Symmetry code: (i) -x + 2,-y - 1,-z; (ii) -x + 1,-y - 1,-z. Hydrogen bonds are shown as dashed lines.

## supplementary crystallographic information

### 1. Comment

In a previous study in our research group, it was possible to obtain the 2-amino-3-(N-oxipiridin-4-ilsulfanil)-propionic acid dihydrate (NPNOCys) by a novel synthesis (Moreno-Fuquen *et al.*, 2010). We then tried to form other organic sulfur compounds by this same synthetic route. In this study nitropyridine N-oxide and 2-mercaptobenzoic acid were combined following the same synthetic route. The crystal structure determination of the title compound (I) was carried out in order to examine its structural characteristics and supramolecular behavior. The molecular structure of the title compound is shown in Fig. 1. Bond lengths (Allen *et al.*, 1987) are in the normal values. The benzene and pyridine rings bridged by the sulfur atom are tilted with respect to each other forming a dihedral angle of 83.93 (7)°. The C—S bond length which links the sulfur to the oxipiridinic ring is similar to the distance in the NPNOCys compound [C—S = 1.7533 (12) Å]. The crystal packing is stabilized by O—H···O hydrogen bonds and weak C—H···O intermolecular interactions, forming *R*_2_^2^(22) and *R*_2_^2^(8) fused-rings along [100] (see Fig. 2; Etter, 1990). The O1 atom of the carboxyl group at (*x*,*y*,*z*) acts as hydrogen-bond donors to O3 atom of the N-oxide group at (-*x* + 1,-*y* + 1,-*z* + 2) and the C11 atom at (*x*,*y*,*z*) acts as hydrogen-bond donors to the O3 atom at (-*x* + 2,-*y* + 1,-*z* + 2) (see Table 1; Nardelli, 1995).

### 2. Experimental

The reagents and solvents for the synthesis were obtained from the Sigma-Aldrich Chemical Co., and they were used without additional purification. To form this compound, equimolar amounts of 4-nitropyridine N-oxide (0.835 g. 5.96 mmol) and thiosalicylic acid were taken. They were completely dissolved in a hot mixture of acetonitrile - methanol (20%) to give a saturated solution. The solution was allowed to slowly evaporating at room temperature. Crystals of good quality and suitable for single-crystal X-ray diffraction were grown from this mixture. The mechanism of the reaction is similar to that reported in other work published before (Moreno-Fuquen *et al.*, 2010).

### 3. Refinement

All H-atoms were placed in calculated positions [C—H= 0.93 Å for aromatic and O—H = 0.82 Å for carboxyl] and they were refined with *U*_iso_(H) 1.2 or 1.5 times *U*_eq_ of the parent atom, respectively.

### Crystal data

**Table:**

C_12_H_9_NO_3_S	*F*(000) = 512
*M**_r_* = 247.26	*D*_x_ = 1.514 Mg m^−^^3^
Monoclinic, *P*2_1_/*c*	Melting point: 505(1) K
Hall symbol: -P 2ybc	Mo *K*α radiation, λ = 0.71073 Å
*a* = 8.9894 (8) Å	Cell parameters from 3414 reflections
*b* = 5.7373 (3) Å	θ = 2.9–27.5°
*c* = 22.5855 (18) Å	µ = 0.29 mm^−^^1^
β = 111.348 (4)°	*T* = 295 K
*V* = 1084.92 (14) Å^3^	Block, pale-yellow
*Z* = 4	0.39 × 0.09 × 0.08 mm

### Data collection

**Table:**

Bruker–Nonius KappaCCD diffractometer	2439 independent reflections
Radiation source: fine-focus sealed tube	1209 reflections with *I* > 2σ(*I*)
Graphite monochromator	*R*_int_ = 0.063
CCD rotation images, thick slices scans	θ_max_ = 27.5°, θ_min_ = 3.6°
Absorption correction: multi-scan (*SADABS*; Sheldrick, 2002)	*h* = −11→8
*T*_min_ = 0.944, *T*_max_ = 0.966	*k* = −6→7
7031 measured reflections	*l* = −29→25

### Refinement

**Table:**

Refinement on *F*^2^	Primary atom site location: structure-invariant direct methods
Least-squares matrix: full	Secondary atom site location: difference Fourier map
*R*[*F*^2^ > 2σ(*F*^2^)] = 0.048	Hydrogen site location: inferred from neighbouring sites
*wR*(*F*^2^) = 0.132	H-atom parameters constrained
*S* = 0.96	*w* = 1/[σ^2^(*F*_o_^2^) + (0.0604*P*)^2^] where *P* = (*F*_o_^2^ + 2*F*_c_^2^)/3
2439 reflections	(Δ/σ)_max_ < 0.001
154 parameters	Δρ_max_ = 0.22 e Å^−^^3^
0 restraints	Δρ_min_ = −0.27 e Å^−^^3^

### Special details

**Table:**

Geometry. All e.s.d.'s (except the e.s.d. in the dihedral angle between two l.s. planes) are estimated using the full covariance matrix. The cell e.s.d.'s are taken into account individually in the estimation of e.s.d.'s in distances, angles and torsion angles; correlations between e.s.d.'s in cell parameters are only used when they are defined by crystal symmetry. An approximate (isotropic) treatment of cell e.s.d.'s is used for estimating e.s.d.'s involving l.s. planes.
Refinement. Refinement of *F*^2^ against ALL reflections. The weighted *R*-factor *wR* and goodness of fit *S* are based on *F*^2^, conventional *R*-factors *R* are based on *F*, with *F* set to zero for negative *F*^2^. The threshold expression of *F*^2^ > σ(*F*^2^) is used only for calculating *R*-factors(gt) *etc*. and is not relevant to the choice of reflections for refinement. *R*-factors based on *F*^2^ are statistically about twice as large as those based on *F*, and *R*- factors based on ALL data will be even larger.

### Fractional atomic coordinates and isotropic or equivalent isotropic displacement parameters (Å^2^)

**Table:**

	*x*	*y*	*z*	*U*_iso_*/*U*_eq_	
S1	0.36646 (9)	0.99831 (11)	0.85995 (4)	0.0459 (3)	
N1	0.8447 (3)	0.8084 (4)	1.00615 (11)	0.0418 (6)	
O3	0.9856 (2)	0.7555 (3)	1.05129 (10)	0.0536 (6)	
C2	0.2227 (3)	0.5798 (4)	0.80591 (13)	0.0404 (7)	
C8	0.5518 (3)	0.9125 (4)	0.91490 (13)	0.0365 (7)	
O2	0.2993 (3)	0.5340 (3)	0.91821 (10)	0.0550 (6)	
C9	0.6268 (4)	1.0653 (5)	0.96478 (14)	0.0424 (7)	
H9	0.5781	1.2055	0.9678	0.051*	
C3	0.3100 (3)	0.7755 (4)	0.80100 (13)	0.0391 (7)	
C12	0.6304 (4)	0.7070 (4)	0.91254 (13)	0.0436 (7)	
H12	0.5838	0.6015	0.8797	0.052*	
O1	0.0548 (3)	0.4332 (4)	0.85447 (10)	0.0701 (7)	
H1	0.0447	0.4003	0.8881	0.105*	
C11	0.7762 (4)	0.6582 (5)	0.95814 (13)	0.0436 (7)	
H11	0.8283	0.5202	0.9559	0.052*	
C1	0.1974 (4)	0.5164 (4)	0.86591 (14)	0.0412 (7)	
C10	0.7716 (3)	1.0091 (5)	1.00914 (14)	0.0449 (7)	
H10	0.8208	1.1123	1.0423	0.054*	
C7	0.1627 (3)	0.4318 (5)	0.75358 (14)	0.0475 (8)	
H7	0.1032	0.3020	0.7559	0.057*	
C4	0.3364 (4)	0.8171 (5)	0.74496 (14)	0.0479 (8)	
H4	0.3944	0.9475	0.7417	0.058*	
C6	0.1904 (4)	0.4752 (5)	0.69837 (15)	0.0547 (9)	
H6	0.1501	0.3743	0.6640	0.066*	
C5	0.2771 (4)	0.6664 (5)	0.69418 (15)	0.0547 (9)	
H5	0.2961	0.6948	0.6570	0.066*	

### Atomic displacement parameters (Å^2^)

**Table:**

	*U*^11^	*U*^22^	*U*^33^	*U*^12^	*U*^13^	*U*^23^
S1	0.0487 (5)	0.0341 (4)	0.0473 (5)	0.0058 (3)	0.0083 (4)	−0.0015 (3)
N1	0.0359 (15)	0.0498 (14)	0.0394 (15)	−0.0031 (11)	0.0133 (12)	−0.0002 (11)
O3	0.0345 (12)	0.0743 (13)	0.0455 (13)	−0.0009 (10)	0.0070 (10)	0.0051 (10)
C2	0.0355 (17)	0.0401 (15)	0.0361 (18)	0.0094 (13)	0.0016 (14)	0.0025 (12)
C8	0.0408 (17)	0.0326 (14)	0.0384 (17)	−0.0036 (12)	0.0170 (14)	0.0023 (11)
O2	0.0559 (15)	0.0636 (13)	0.0380 (13)	−0.0129 (11)	0.0082 (11)	−0.0030 (10)
C9	0.0426 (19)	0.0406 (15)	0.0464 (19)	−0.0015 (13)	0.0192 (16)	−0.0058 (13)
C3	0.0358 (17)	0.0357 (15)	0.0392 (18)	0.0079 (12)	0.0057 (14)	0.0021 (12)
C12	0.048 (2)	0.0387 (16)	0.0397 (18)	−0.0008 (13)	0.0112 (16)	−0.0040 (12)
O1	0.0446 (15)	0.1016 (18)	0.0569 (16)	−0.0104 (13)	0.0099 (12)	0.0222 (12)
C11	0.0444 (19)	0.0396 (16)	0.0450 (19)	0.0040 (13)	0.0141 (16)	0.0000 (13)
C1	0.0393 (19)	0.0321 (14)	0.0475 (19)	0.0039 (13)	0.0103 (15)	0.0017 (12)
C10	0.0448 (19)	0.0461 (17)	0.0455 (19)	−0.0097 (15)	0.0186 (16)	−0.0086 (13)
C7	0.0404 (19)	0.0429 (16)	0.047 (2)	0.0015 (13)	0.0015 (16)	−0.0013 (13)
C4	0.042 (2)	0.0533 (18)	0.044 (2)	0.0037 (14)	0.0105 (16)	0.0047 (14)
C6	0.053 (2)	0.062 (2)	0.039 (2)	0.0083 (16)	0.0039 (16)	−0.0118 (15)
C5	0.057 (2)	0.064 (2)	0.041 (2)	0.0121 (17)	0.0157 (17)	0.0040 (15)

### Geometric parameters (Å, º)

**Table:**

S1—C8	1.747 (3)	C12—C11	1.369 (4)
S1—C3	1.781 (3)	C12—H12	0.9300
N1—C10	1.339 (3)	O1—C1	1.302 (3)
N1—O3	1.341 (3)	O1—H1	0.8200
N1—C11	1.346 (3)	C11—H11	0.9300
C2—C7	1.395 (4)	C10—H10	0.9300
C2—C3	1.397 (4)	C7—C6	1.380 (4)
C2—C1	1.498 (4)	C7—H7	0.9300
C8—C12	1.385 (4)	C4—C5	1.379 (4)
C8—C9	1.393 (4)	C4—H4	0.9300
O2—C1	1.207 (3)	C6—C5	1.369 (4)
C9—C10	1.361 (4)	C6—H6	0.9300
C9—H9	0.9300	C5—H5	0.9300
C3—C4	1.391 (4)		
			
C8—S1—C3	105.49 (12)	N1—C11—H11	119.8
C10—N1—O3	120.1 (2)	C12—C11—H11	119.8
C10—N1—C11	120.2 (3)	O2—C1—O1	124.5 (3)
O3—N1—C11	119.7 (2)	O2—C1—C2	123.6 (3)
C7—C2—C3	118.5 (3)	O1—C1—C2	111.8 (3)
C7—C2—C1	118.7 (3)	N1—C10—C9	121.5 (3)
C3—C2—C1	122.8 (2)	N1—C10—H10	119.3
C12—C8—C9	117.6 (3)	C9—C10—H10	119.3
C12—C8—S1	125.5 (2)	C6—C7—C2	121.0 (3)
C9—C8—S1	116.9 (2)	C6—C7—H7	119.5
C10—C9—C8	119.9 (3)	C2—C7—H7	119.5
C10—C9—H9	120.1	C5—C4—C3	120.5 (3)
C8—C9—H9	120.1	C5—C4—H4	119.8
C4—C3—C2	119.8 (2)	C3—C4—H4	119.8
C4—C3—S1	117.4 (2)	C5—C6—C7	120.1 (3)
C2—C3—S1	122.2 (2)	C5—C6—H6	120.0
C11—C12—C8	120.5 (3)	C7—C6—H6	120.0
C11—C12—H12	119.8	C6—C5—C4	120.2 (3)
C8—C12—H12	119.8	C6—C5—H5	119.9
C1—O1—H1	109.5	C4—C5—H5	119.9
N1—C11—C12	120.4 (3)		
			
C3—S1—C8—C12	−2.5 (3)	C7—C2—C1—O2	137.2 (3)
C3—S1—C8—C9	176.8 (2)	C3—C2—C1—O2	−39.8 (4)
C12—C8—C9—C10	−0.5 (4)	C7—C2—C1—O1	−40.6 (3)
S1—C8—C9—C10	−179.9 (2)	C3—C2—C1—O1	142.3 (3)
C7—C2—C3—C4	−0.6 (4)	O3—N1—C10—C9	−178.6 (2)
C1—C2—C3—C4	176.4 (2)	C11—N1—C10—C9	0.8 (4)
C7—C2—C3—S1	170.4 (2)	C8—C9—C10—N1	−0.1 (4)
C1—C2—C3—S1	−12.5 (4)	C3—C2—C7—C6	0.8 (4)
C8—S1—C3—C4	−99.3 (2)	C1—C2—C7—C6	−176.3 (3)
C8—S1—C3—C2	89.5 (2)	C2—C3—C4—C5	−0.1 (4)
C9—C8—C12—C11	0.3 (4)	S1—C3—C4—C5	−171.6 (2)
S1—C8—C12—C11	179.6 (2)	C2—C7—C6—C5	−0.4 (4)
C10—N1—C11—C12	−1.0 (4)	C7—C6—C5—C4	−0.4 (5)
O3—N1—C11—C12	178.4 (2)	C3—C4—C5—C6	0.6 (5)
C8—C12—C11—N1	0.5 (4)		

### Hydrogen-bond geometry (Å, º)

**Table:**

*D*—H···*A*	*D*—H	H···*A*	*D*···*A*	*D*—H···*A*
C11—H11···O3^i^	0.93	2.35	3.255 (3)	164
O1—H1···O3^ii^	0.82	1.74	2.530 (3)	162

Symmetry codes: (i) −*x*+2, −*y*+1, −*z*+2; (ii) −*x*+1, −*y*+1, −*z*+2.
